# Phosphoproteomic Landscape of HDLBP: Insights into Function and Disease Associations

**DOI:** 10.3390/ijms27052147

**Published:** 2026-02-25

**Authors:** Pathiyil Sajini Sekhar, Amal Fahma, Suhail Subair, Leona Dcunha, Althaf Mahin, Athira Perunally Gopalakrishnan, Rajesh Raju, Sowmya Soman

**Affiliations:** Centre for Integrative Omics Data Science (CIODS), Yenepoya (Deemed to be University), Mangalore 575018, Karnataka, India; sajinisekhar333@gmail.com (P.S.S.); amalfahma7@gmail.com (A.F.); suhailrejeena007@gmail.com (S.S.); leonadcunha@gmail.com (L.D.); althafmahin99@gmail.com (A.M.); athirajrf@yenepoya.edu.in (A.P.G.)

**Keywords:** HDLBP, phosphoproteomics, phosphosites, co-regulation, cancer

## Abstract

High-density lipoprotein-binding protein (HDLBP), also called Vigilin, is a multifunctional RNA-binding protein with established roles in RNA transport and regulation, chromosome segregation, lipid homeostasis, and translational regulation. Frequently detected to be perturbed in phosphoproteome analysis, phosphorylation is indicated as a major mechanism in the regulation of HDLBP functions; however, its phosphorylation landscape remains unexplored. We performed a meta-phosphoproteome analysis of HDLBP to map site-specific functional and regulatory roles of its two most frequently detected phosphosites, S31 and S944. Co-occurrence analysis across multiple datasets indicated that they can be phosphorylated together, suggesting potential co-ordinated regulation. Site-specific co-regulation analysis revealed distinct phospho-regulatory networks, with upstream kinases identified exclusively for S944. Functional enrichment of co-regulated protein phosphosites (CPPs) highlighted its role in RNA metabolism, chromosome organization, and nucleoplasmic transport, while functional annotation of site-specific phosphorylation of CPPs indicates its involvement in cell cycle regulation, apoptosis, and carcinogenesis. Additionally, the potential role of CPPs in the lipid homeostasis network was explored. Furthermore, the differential expression of HDLBP phosphosites across multiple cancers was observed using UALCAN, suggesting a potential role for phospho-regulation of HDLBP in tumor-associated pathways. Together, these findings provide the first integrated view of HDLBP phosphorylation and could serve as a valuable framework for future targeted studies to elucidate the mechanistic roles of site-specific HDLBP phosphorylation in cellular and pathophysiological processes.

## 1. Introduction

In modern systems biology, large-scale analyses of post-translational modifications (PTMs) are essential for decoding complex cellular signaling cascades. Among the different types of PTMs, reversible protein phosphorylation mediated by kinases and phosphatases is a key regulator of cellular processes and intracellular signaling pathways [1]. Over the past two decades, technological advances in this field have enabled considerable quantitative mapping of organization and regulation of cellular signaling networks [2]. Phosphoproteomics enables large-scale and relatively unbiased mapping of phosphorylation events, primarily through mass spectrometry (MS)-based approaches [3,4]. The dysregulation of phosphorylation-dependent signaling cascades is frequently associated with human diseases, including cancer, metabolic disorders, viral infections, and neurodegenerative conditions [1,5,6]. Although large-scale phosphoproteomics studies have catalogued thousands of phosphosites, very few among them have their functions explored [7]. This has resulted in a large number of dark phosphosites in the human proteome. Thus, a systematic phosphoproteomic analysis provides a unique and dynamic perspective on how cellular physiology is orchestrated at the molecular level.

High-density lipoprotein binding protein (HDLBP), also known as Vigilin, is a highly conserved RNA-binding protein (RBP) first characterized by Graham et al., in 1987 [8]. The HDLBP gene located on the long arm (q arm) of human chromosome 2, specifically at 2q37.3, is ubiquitously expressed and its localization is reported in cytosol, nucleus, and endoplasmic reticulum [9,10,11]. HDLBP is composed of 1268 amino acid residues with 141,440 Da [12,13]. Until 1992, it was considered as a possible binding partner of high-density lipoprotein (HDL) [14]. It contains multiple sequence repeats that function as nucleic acid–binding domains similar to the K homology (KH) domains found in heterogeneous nuclear RNA-binding proteins (hnRNPs) [15]. The name ”Vigilin” became commonly used for HDLBP since it is derived from an N-terminal motif VIG (valine–isoleucine–glycine) [16]. These proteins are characterized by 14 KH domains that cover most of the protein’s sequence. In higher eukaryotes, an additional KH domain has been detected in the N terminus [17]. The KH domain of HDLBP possesses a high affinity for the CU-rich 3’ untranslated region of mRNAs, thus influencing its stability, localization, and translation efficiency [18,19].

The multifunctional role of HDLBP is implicated in RNA transport, translational regulation, heterochromatin-mediated gene silencing, chromosome segregation, cytoskeleton organization, DNA repair, and cholesterol transport [18,20]. In the nucleus, HDLBP interacts with the histone methyltransferase SUV39H1 and contributes to heterochromatin organization and RNA-dependent gene silencing. It has also been implicated in maintaining genome stability through roles in DNA double-strand break repair and chromatin-associated regulatory processes [20,21]. HDLBP promotes glycolysis and CD8^+^ T-cell exhaustion in lung adenocarcinoma by binding to and stabilizing GJB2 mRNA, thereby driving tumor progression [22]. HDLBP regulates cholesterol metabolism by linking HDL/apoA-I signaling to RNA-mediated control of cholesterol transport. The movement of cholesterol inside cells is regulated by HDLBP by binding to RNA and interacting with HDL and apoA-I [23]. Besides its role in cholesterol transport, HDLBP controls lipid metabolism by modulating the translation of mRNAs in the liver that code for secretory and proatherogenic proteins, including apoB and apoC-III. By binding CU-rich regions in the mRNAs of apoB and other proatherogenic secreted proteins, HDLBP promotes triglyceride metabolism and VLDL secretion thus influencing lipid release from the liver, thereby contributing to dyslipidemia [24]. HDLBP interaction with ER-targeted mRNAs enhanced the translation of secretory and transmembrane proteins [19]. Attributed to these diverse cellular roles, unsurprisingly, HDLBP has been well associated with influencing the severity and progression of multiple diseases. It is frequently reported to be upregulated in various cancers to promote tumor growth and metastasis such as in breast cancer and hepatacellular carcinoma (HCC) [25,26]. In HCCs, HDLBP serves as a critical mediator by stabilizing and maintaining the activity of RAF1 thereby promoting HCC proliferation and sorafenib resistance [27,28]. In atherosclerotic plaques, HDLBP was particularly enriched in foam cell macrophages and co-localized with the lipid transport protein apolipoprotein E (ApoE) well implicated in atherosclerosis [29]. Knockdown of HDLBP in the liver decreases the formation of atherosclerotic plaques by reducing LDL/VLDL levels, suggesting the role of HDLBP in liver metabolism [24]. Additionally, HDLBP is also found upregulated in patients with non-alcoholic fatty liver disease [24]. Felder et al., 2009 reported HDLBP as an autism-associated gene, as the lower levels of HDLBP expression attributed to its microdeletion of 2q37.3 loci (brachydactyly–mental retardation syndrome) exhibited neurodevelopmental delay found in autism spectrum disorders [30].

HDLBP binding to certain viral RNAs is shown to facilitate viral replication, particularly, in single-stranded plus-sense RNA viruses, including flaviviridae, caliciviridae, or SARS-CoV19 [31,32,33,34]. Furthermore, HDLBP and SERBP1 (SERPINE1 mRNA-Binding Protein 1) can be recruited to 40S ribosomal subunit by RACK1 to facilitate its interaction with viral RNA and host translational machinery in DENV infection. Silencing HDLBP significantly reduced viral replication and envisages it as a promising target for antiviral therapies [31,35]. Hence, HDLBP is considerably a key regulatory hub of lipid metabolism, RNA regulation in oncogenesis, and viral replication and embraces it as a potential biomarker and therapeutic target in these pathological conditions.

Despite the envisioned array of protein-level functions, the post-translational modifications that regulate these functions remain poorly explored. Considering the involvement of HDLBP in diverse biological processes, exploring the phosphoproteomic landscape of HDLBP offers a unique opportunity to uncover how phosphorylation governs its activity and mediates disease-related signaling pathways. Thus, a systematic analysis of HDLBP phosphorylation could reveal novel regulatory motifs, functional phosphorylation sites, and context-specific signaling networks that are otherwise masked in conventional proteomic studies. So, in this study, we present a comprehensive global phosphoproteomic analysis of HDLBP to delineate its phosphorylation dynamics, functional relevance, and disease associations. By integrating large-scale phosphosite mapping with bioinformatic network analysis, we explored the possible signaling connections and identified the potential disease-associated phosphorylation events, thereby expanding our current understanding of HDLBP. Our findings not only provide a foundational resource for the HDLBP research community but also underscore the broader utility of phosphoproteomics in decoding the regulatory mechanisms of multifunctional proteins implicated in health and disease, offering new opportunities for targeted studies and therapeutic interventions.

## 2. Results

### 2.1. Assembly and Analysis of Global Phosphoproteomic Data of HDLBP

To identify functionally relevant phosphosites, we systematically screened over 3825 publicly available human global phosphoproteomic datasets, resulting in the identification of 932 profiling datasets and 194 differential datasets containing class-1 HDLBP phosphosites. After the extensive mapping of class-1 phosphosites across these datasets, 39 distinct phosphosites were identified in profiling, and 21 were found to be differentially regulated across various experimental conditions, including cancers, infections, and hormone stimulation of cells. The profiling and differential datasets of class-1 phosphosites of HDLBP are given in Supplementary Materials S1A,B.

### 2.2. Identification of Predominantly Detected Phosphosites of HDLBP

**Figure 1:**
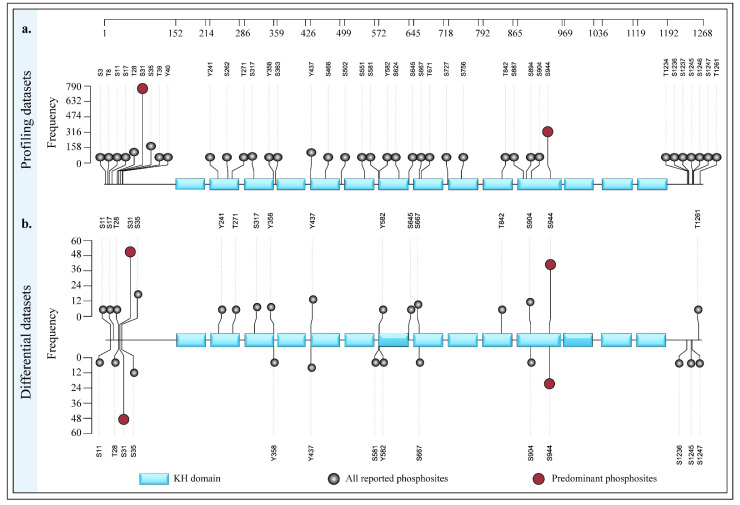
Lollipop plot visualization of phosphosites within HDLBP with their detection frequencies in global cellular phosphoproteomics datasets. (**a**) Phosphosites detected in the qualitative profiling datasets (**b**) Phosphosites detected in the quantitative differential datasets.

The HDLBP phosphosites were detected from diverse experimental/biological conditions, so we ranked the frequency of detection of each phosphosite across different datasets. Interestingly, the detected phosphosites of HDLBP varied widely in frequency. Since frequently detected phosphosites are more likely to be functionally relevant, we focused on those with the highest detection frequency. Among the 21 differentially regulated phosphosites, S31 and S944 were identified as the predominant phosphosites across 103 profile and 65 differential analysis experimental conditions, respectively. The predominant phosphosite S31 was located outside the functional domain of HDLBP, while S944 is located in a KH domain of HDLBP. The frequency of all 39 HDLBP phosphosites in qualitative profiling (Figure 1a) and 21 in quantitative differential datasets (Figure 1b) is represented in the lollipop plot.

### 2.3. Sequence Coverage of HDLBP Protein

HDLBP peptides compiled from multiple mass spectrometry-based datasets were mapped to the protein sequence to delineate key regulatory regions, including RNA-binding domains, and to confirm previously reported phosphorylation sites. Moreover, increased sequence coverage allows for more accurate mapping of post-translational modifications (PTMs), improves the ability to differentiate between protein isoforms, enhances quantitative proteomics, and offers deeper insights into protein structure [36,37].

**Figure 2:**
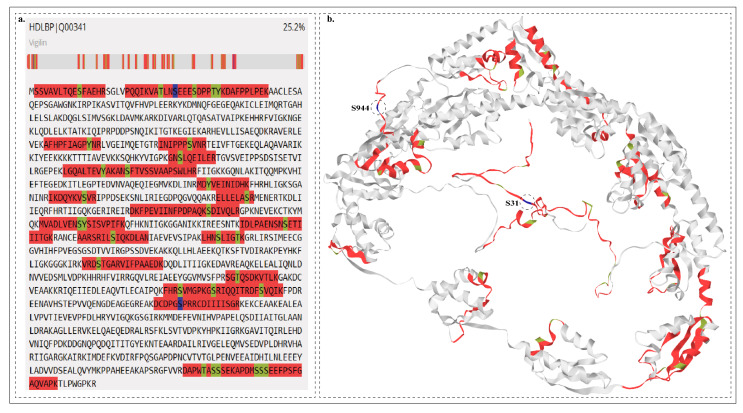
The sequence coverage and the class-1 phosphosites identified from 932 HDLBP phosphoproteomic datasets. The green represents the identified HDLBP phosphosites, blue represents predominant HDLBP phosphosites, and red indicates total sequence coverage. (**a**) A sequence coverage of 25.2% was achieved, corresponding to the 1268-amino-acid sequence of HDLBP. (**b**) The 3D-predicted HDLBP model shows peptides identified in profiling data.

The analysis focused on the modified peptide sequences identified from the human phosphoproteomic profile datasets. The percentage of sequence coverage was determined by assessing the ratio of total observed protein sequence length to total protein sequence length. The analysis of 932 global profile datasets identified 39 peptides corresponding to the HDLBP protein. These peptides covered 25.2% of the full 1268-amino-acid sequence of HDLBP. This level of sequence coverage highlights the portion of the protein that is high-flying and detectable by mass spectrometry-based phosphoproteomics. The coverage and corresponding structural representation were visualized using the Sequence Coverage Visualizer (SCV) tool and are presented in Figure 2.

### 2.4. Analysis of Phosphoproteins and Phosphosites Co-Differentially Regulated with Predominant HDLBP Sites

To explore the function and regulation of HDLBP at the phosphosite-centric network level, a co-regulation analysis was employed to filter co-regulated phosphosites in other proteins (CPPs) that consistently show co-differential expression with the predominant phosphosites of HDLBP. Towards this, we computed and ranked the CPPs that show upregulation or downregulation relative to the HDLBP’s predominant sites. Prominent phosphosites with positive or negative co-regulation were filtered using stringent inclusion and exclusion criteria (detailed in the Materials and Methods Section 4) to ensure the consistency of co-regulation patterns and to minimize potential dataset biases. This analysis revealed 542 positively co-regulated and 17 negatively co-regulated high-confidence CPPs with the HDLBP S31 predominant site, and 1123 positively co-regulated and 70 negatively co-regulated high-confidence CPPs with the S944 predominant site of HDLBP (Supplementary Materials S2).

**Figure 3:**
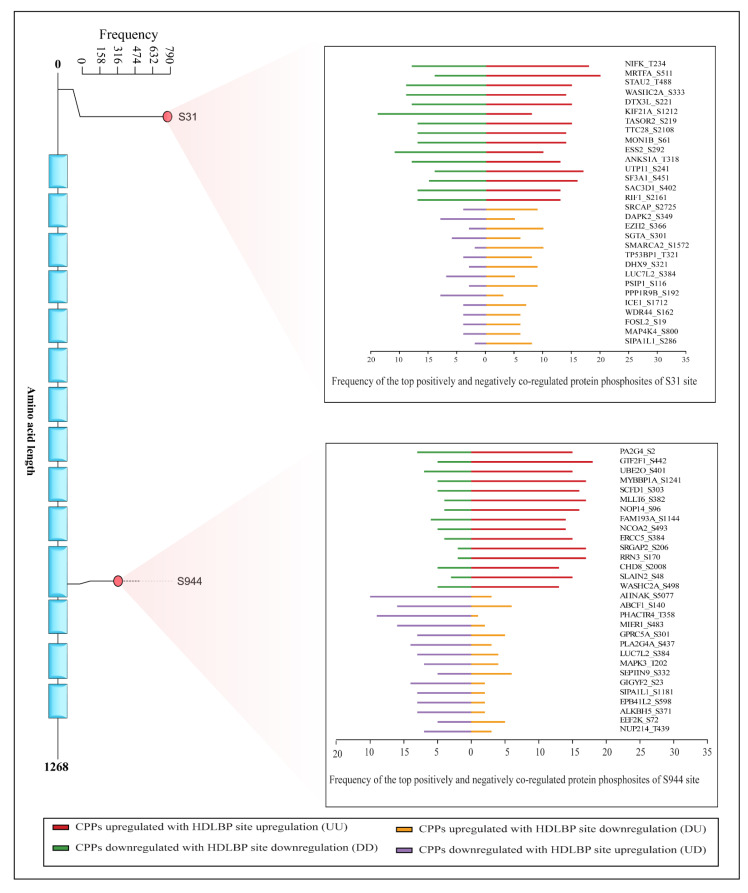
Bar plot representing the top co-regulated proteins (CPPs) of respective predominant site S31 located outside the domain and S944 located inside the domain.

The top positively co-regulated CPPs of the HDLBP S31 site include NIFK_T234, MRTFA_S511, and STAU2_T488, with frequencies of 31, 29, and 29, respectively. The top negatively co-regulated CPPs include SRCAP_S2725, DAPK2_S349, and EZH2_S366, with a frequency of 13 for each phosphosite. Similarly, for the S944 site, the top positive CPPs include PA2G4_S2, GTF2F1_S442, and UBE2O_S401, with frequencies of 23, 23, and 22, and the negative CPPs include AHNAK_S5077, ABCF1_S140, and PHACTR4_T358, with frequencies of 18, 17, and 15, respectively. The majority of these co-regulated phosphosites lack defined molecular or functional characterization. Figure 3 illustrates the top positively and negatively co-regulated CPPs.

### 2.5. Co-Occurrence Analysis of HDLBP Phosphosites

**Figure 4:**
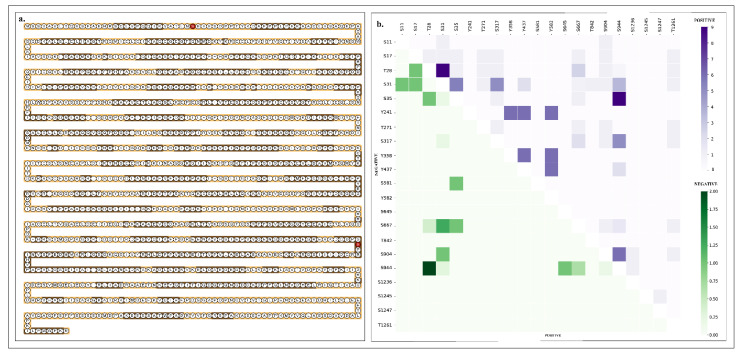
(**a**) Visualization of tryptic peptides within HDLBP identified using the ExPASy PeptideCutter tool, with phosphosites in HDLBP highlighted in red. (**b**) Co-occurrence analysis of HDLBP phosphosites based on expression co-regulation. The heat map represents the co-occurrence pattern of phosphosites, where violet indicates positive co-regulation, while green represents negative co-regulation between phosphosite pairs of green, with a color gradient representing the frequency of co-regulation.

Phosphorylation sites that co-occur often tend to exhibit functional similarity and are commonly conserved through evolution [38]. This suggests that such sites together contribute to a particular biological function, localization, or interaction [39]. To explore this in the context of HDLBP, we analyzed the co-occurrence patterns of differential expression of its phosphosites. Interestingly, the predominant phosphosite S944 exhibited a strong co-occurrence pattern with S35 across nine differential datasets and with S31 across six datasets. This highlights the potential site-level co-ordination and functional interplay within HDLBP’s phosphorylation landscape. The observed co-regulatory relationships between HDLBP phosphorylation sites are visualized in Figure 4, and the co-occurrence data are given in Supplementary Materials S3.

### 2.6. Analysis of Co-Regulated Phosphosites in Kinases and Phosphatases

**Figure 5:**
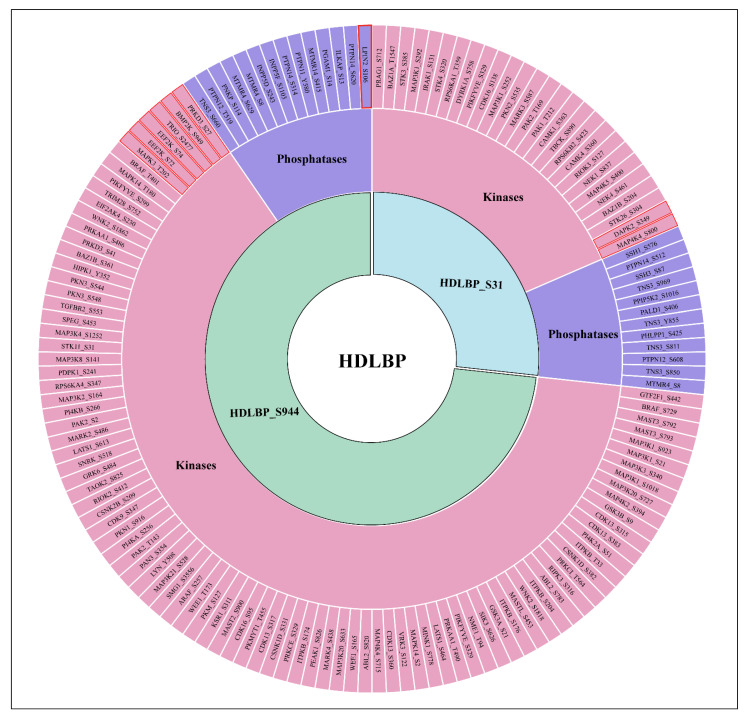
Circular diagram showing the co-regulated kinases and phosphatases. Color coding in the figure indicates kinases in pink and phosphatases in purple, while inner ring segments in light blue and light green correspond to HDLBP_S31 and HDLBP_S944, respectively.CPPs with predominant phosphosites and activity-associated sites marked in a red box.

Protein kinases and phosphatases act as molecular switches, turning protein activity on or off through phosphorylation and dephosphorylation [40]. At the S31 site, 25 CPPs identified as kinases and 12 as phosphatases showed positive co-regulation, whereas only 2 kinase CPPs exhibited negative co-regulation. In contrast, for the S944 site, 87 kinase CPPs and 13 phosphatase CPPs were positively co-regulated, while 6 kinases and 1 phosphatase exhibited negative co-regulation. The list of kinase CPPs and phosphatase CPPs are given in Figure 5 and Supplementary Materials S4A.

The co-regulated network is mainly composed of kinases and this likely reflects the role in different co-ordinated signaling cascades. These CPPs span MAPK, AGC, CAMK, NEK, and related families, implicating pathways that control cell growth, stress responses, cytoskeletal dynamics, and membrane trafficking. Phosphorylation at the identified positions is therefore likely to modulate kinase activity, substrate selection, or subcellular localization, making these sites high-priority candidates for targeted validation.

### 2.7. Potential Upstream Kinases of Predominant HDLBP Phosphosites

Upstream kinases are key modulators involved in the regulation of cellular signaling by phosphorylation of a target protein. CHK1 is a putative kinase identified so far to be associated with HDLBP phosphorylation [41]. Thus, to identify potential upstream kinases associated with HDLBP predominant sites, we used the kinase-substrate motif specificity analysis based on the data derived from Johnson et al. [42]. Additionally, computational tools such as NetworKIN, AKID, and iKiP-DB were used to predict the potential upstream kinases of HDLBP.

**Figure 6:**
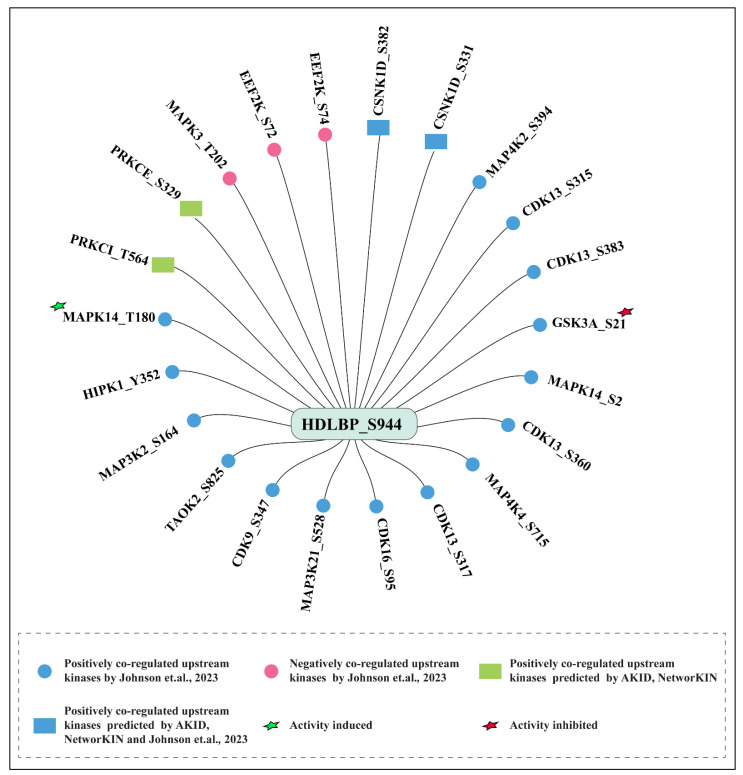
The upstream kinases are shown in the circular dendrogram [42]. The activity-induced site is marked in green and the inhibited site is marked in red.

Upstream kinases were identified exclusively for the S944 site. Among these, 17 kinases were reported by Johnson et al., and 4 additional kinases were predicted through in silico analysis, all showing positive co-regulation. Among these, previously, we proposed Y352 as a functionally relevant phosphosite in HIPK1 [43]. In contrast, 3 upstream kinases identified by Johnson et al. study exhibited negative co-regulation [42]. The predicted upstream kinases are given in Figure 6 and Supplementary Materials S4B.

### 2.8. Identification of Protein and Phosphosite-Specific Interactors of HDLBP

**Figure 7:**
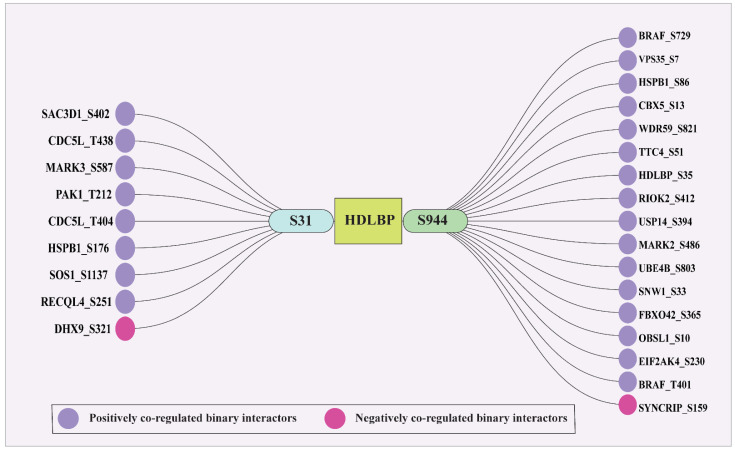
Phosphosites in known binary interactors that co-regulated with predominant HDLBP phosphosites are shown, where purple denotes positively co-regulated interactors and pink denotes negatively co-regulated interactors.

The binary and complex interactors of HDLBP were retrieved from various databases like HPRD, BIND, BioGRID, ConsensusPathDb, CORUM, and RegPhos. The list of binary partners and complex interactors are given in the Supplementary Materials S4C,D. We identified eight binary interactors that are positively co-regulated and one interactor which is negatively co-regulated for the S31 predominant site. Similarly, we identified 16 positive and 1 negative co-regulated binary interactor of S944. Figure 7 shows the co-regulated binary interactors.

### 2.9. Inferring the Biological Process Associated with HDLBP-Predominant Sites

To gain insights into the biological processes associated with HDLBP, gene enrichment analyses of CPPs were performed using ShinyGO. It enables us to identify the potential pathways and cellular activities influenced by its regulation. The major enriched processes revealed that the input gene set is significantly enriched in biological processes related to chromatin remodeling, cell cycle regulation, RNA metabolism, and nucleocytoplasmic transport, suggesting the role of HDLBP in gene regulation, nuclear dynamics, and cytoskeletal organization. The gene enrichment analysis data is given in Supplementary Materials S5.

**Figure 8:**
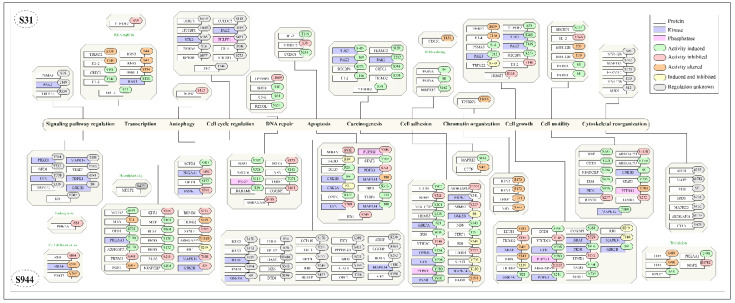
The phosphosite-specific biological processes of the co-regulated protein phosphosites.

Analysis of HDLBP at the phosphosite-specific level helped us to identify the in-depth regulatory role, including the activation (“on”) or inhibition (“off”) of specific molecular functions. This enabled us to understand how individual phosphosites can control protein activity and affect different cellular processes. The identified phosphosites include both activity-induced and activity-inhibited phosphorylation events, as well as sites exhibiting altered activity (altered function without induction or inhibition). Through functional mapping, the co-regulated proteins were found to participate in diverse cellular processes, including regulation of the cell cycle, apoptosis, inhibition of autophagy, transcriptional control, DNA repair mechanisms, modulation of signaling pathways, and reorganization of the cytoskeleton (Figure 8). From Figure 8, it can be observed that comparatively more CPPs are associated with cell cycle regulation, carcinogenesis, and transcription-related processes. In cell cycle regulation, CPPs associated with the S31 phosphosite include UHRF1_S639, TP53BP1_T1609, STK3_S385, PSMA5_S16, RPTOR_S877, CUEDC2_S110, PAK2_T169, PHLPP1_S425, H1-4_T146, SHCBP1_S273, and H1-2_T146. The CPPs associated with the S944 phosphosite include RRN3_S170, RRN3_S172, BRAF_S729, FADD_S194, GSK3B_S9, CDC6_S54, RPL12_S38, TIAM1_S1466, NBN_S343, DTD1_S196, CCP110_S170, TERF1_T371, OPTN_S177, RB1_S249, HJURP_S473, EIF5_S389, PPP1R12A_S473, LIMD1_S272, STAT3_Y705, ORC2_T116, ATRIP_S239, CGGBP1_S164, BORA_S252, MAPK14_T180, and EIF5_S390. Other functional categories containing site-specific CPPs include apoptosis, autophagy, carcinogenesis, cell adhesion, cell differentiation, cell growth, cell motility, chromatin organization, cytoskeletal reorganization, DNA repair, endocytosis, neural plasticity, signaling pathway regulation, transcription, translation, RNA splicing, and RNA stability, indicating that HDLBP phosphorylation is associated with diverse cellular regulatory processes.

### 2.10. HDLBP Phospho-Network in Lipid Homeostasis

Thus far, our analysis revealed that several protein phosphosites co-regulated with the predominant phosphorylation sites of HDLBP. Since HDLBP is known to play a role in lipid metabolism and cholesterol transport, we analyzed various publicly available domains and databases, such as Reactome and AmiGO, to evaluate the phosphoproteins co-regulated with HDLBP sites.

For the HDLBP_S31 site, CPPs included EP300_S176, CRTC3_S391, CRTC2_S136, FASN_S725, SEC14L1_T234, SORT1_S825, SNX17_S409, SNX17_S336, SNX17_S359, BLTP1_S1355, and PNPLA6_S354. Many of these proteins were reported to be involved in lipid biosynthesis, vesicular trafficking, and lipoprotein sorting, supporting the potential involvement of HDLBP in intracellular lipid transport and metabolic signaling.

**Figure 9:**
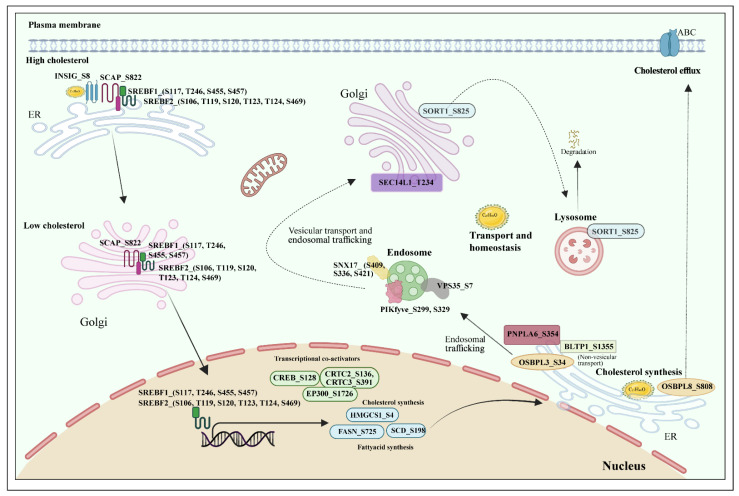
Schematic representation of lipid homeostasis highlighting CPPs involved in sensing, synthesis, trafficking, and efflux, which are co-regulated with the predominant sites.

Similarly, for the HDLBP_S944 site, CPPs such as SCAP_S822, HMGCS1_S4, SCD_S198, OSBPL3_S265, OSBPL3_S34, OSBPL8_S808, OSBPL11_T27, CREB1_S128, VPS35_S7, and PIKFYVE_S299/S329 were identified to be co-regulated. Several of these proteins, including SCAP, HMGCS1, and SCD, are central components of cholesterol biosynthesis and lipid regulatory pathways, further reinforcing a functional association between HDLBP phosphorylation and lipid homeostasis. Figure 9 presents the related proteins from our co-regulated list that are involved in lipid homeostasis. Interestingly, all of these co-regulated phosphosites were also observed to be perturbed by metformin, suggesting that HDLBP phosphorylation may be responsive to metabolic cues modulated by this drug.

### 2.11. HDLBP in Different Cancers

Based on the University of Alabama at Birmingham Cancer (UALCAN) datasets, the expression of the predominant phosphorylation sites of HDLBP were found differentially regulated in carcinomas of breast, colon, pancreas, and in glioblastoma, and hepatocellular carcinoma (HCC). The z-score-based analysis reflects the standard deviation from the median protein expression across tumor and normal samples for each cancer type.

**Figure 10:**
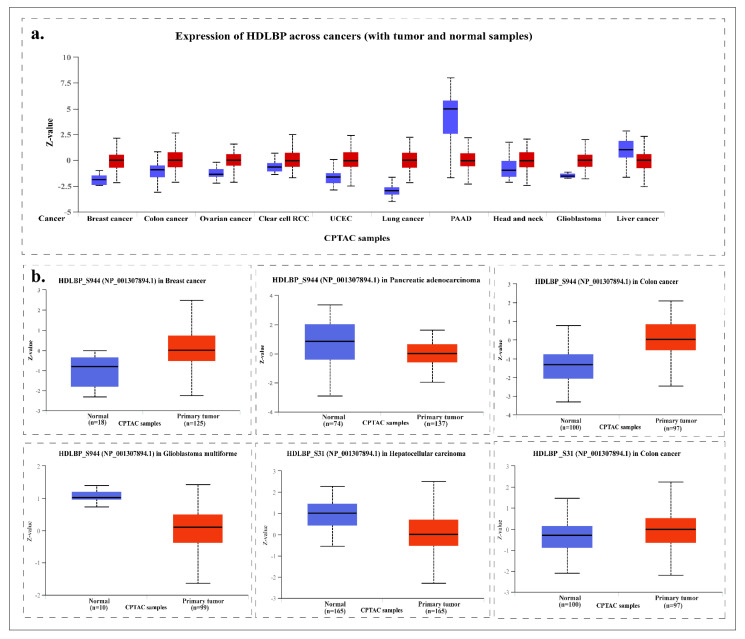
(**a**) Pan-cancer analysis of the expression level of HDLBP. (**b**) Box plot depicting the predominant site-specific expression of HDLBP in different cancers. Blue boxes represent normal samples, and red boxes represent primary tumor samples across the indicated cancer types.

Protein expression levels were estimated based on the distribution of expression values within the analyzed population. A *p*-value < 0.05 was considered statistically significant, indicating that HDLBP showed differential expression during tumor progression. Detailed results are presented in Table 1 and illustrated in Figure 10. The observed variation in HDLBP phosphorylation across different cancer types indicates that its regulation is tumor-context dependent rather than uniform across malignancies. Specifically, the S944 phosphosite shows increased levels in breast and colon cancers, while S31 phosphorylation is also upregulated in colon cancer. In contrast, S944 phosphorylation is downregulated in pancreatic cancer, glioblastoma, and HCC. These findings suggest that HDLBP phosphorylation may contribute differently to tumor-associated regulatory networks depending on cancer type and cellular context.

## 3. Discussion

HDLBP, a large, multifunctional RNA-binding protein, plays a crucial role in regulating post-transcriptional gene expression by contributing to various aspects of RNA metabolism and transport [20]. Despite growing insights into HDLBP’s protein-level functions, the biological implications of its site-specific phosphorylation are still largely unexplored, leaving a major gap in understanding how its function is modulated through phosphorylation [18]. Investigating the phosphoproteomic landscape of HDLBP is essential to understand how site-specific phosphorylation regulates its RNA-binding activity, subcellular localization, and roles in gene regulation. As no prior phosphoproteomic analysis of HDLBP exists, we performed a comprehensive phosphoproteomic analysis to identify predominantly detected phosphosites and assess their potential regulatory functions.

Interestingly, the predominant site S31 was located in the N-terminal region outside the functional domain, while the other predominant S944 was located within the C-terminal KH domain. The N- and C-terminal regions of HDLBP serve distinct functions, with the N-terminus containing putative nuclear localization (NLS) and export (NES) signals, while the C-terminus contains the majority of the RNA-binding sites [44,45,46,47]. Although these phosphorylation sites are positioned at opposite ends of the protein, they frequently co-occur under multiple experimental conditions, as highlighted in the co-occurrence analysis presented in the results. This suggests that both sites may participate in the same functional or regulatory pathway, as the co-occurrence of phosphorylation sites is often linked to functional similarity and evolutionary conservation [38,48]. Given the lack of prior phosphoproteomic studies on HDLBP, a sequence coverage visualization was generated to illustrate the distribution of identified phosphorylation sites. Sequence coverage is an important parameter in bottom-up proteomics, representing the proportion of the identified peptide sequence length relative to the entire protein sequence length [36,49]. Sequence coverage analysis revealed 25.2% coverage of the 1268–amino acid HDLBP sequence.

A subsequent co-regulation analysis of CPPs was performed to further understand functional roles and associated pathways. Among the co-regulated CPPs, NIFK_T234 and PA2G4_S2 were the top positively co-regulated CPPs with S31 and S944, respectively, whereas SRCAP_S2725 and AHNAK_S5077 were the top negatively co-regulated CPPs. Nucleolar protein interacting with the FHA domain of pKI-67 (NIFK) is a nucleolar protein that binds Ki-67 via phosphorylation at T234, regulating ribosome biogenesis, mitotic progression, and cancer metastasis [50], whereas, proliferation-associated protein 2G4 (PA2G4), also known as ErbB3-binding protein 1 (EBP1), is a multifunctional RNA- and protein-binding regulator involved in transcription, translation, and cancer signaling. The phosphorylation of PA2G4 modulates protein interactions and subcellular localization that affect proliferation and oncogenesis [51]. The negatively co-regulated protein phosphosites Snf2 Related CREBBP Activator Protein (SRCAP) is a chromatin-remodeling ATPase that activates transcription via histone exchange, and phosphorylation at serine residues modulates its role in DNA repair and transcriptional regulation, influencing genomic stability and development [52]. Similarly, Neuroblast Differentiation-Associated Protein (AHNAK) was negatively co-regulated, reflecting its potential role in stabilizing cytoskeletal structures and regulating membrane-associated signaling complexes [53].

Further, our phosphocentric analysis revealed that a significant proportion of CPPs were protein kinases, highlighting the presence of strong signaling signatures within the HDLBP regulatory network. Using kinase–substrate predictions from Johnson et al. [42] and additional in silico tools, we identified a set of upstream kinases that mapped exclusively to the S944 site. The upstream kinases such as CDK13 and CDK9 primarily regulate transcriptional elongation and mRNA processing via phosphorylation of the RNA Polymerase II C-terminal domain, ensuring proper gene expression and RNA maturation [54,55,56]. CDK16 regulates vesicle trafficking and cytoskeletal dynamics, with emerging roles in cell cycle progression and RNA-associated processes [57]. The MAPK and MAP3K cascades (MAPK14, MAP3K2, MAP3K21, MAP4K4) integrate oxidative and inflammatory stimuli to trigger stress responses, apoptosis, and cytoskeletal reorganization, connecting external stress to transcriptional regulation [58,59]. HIPK1 (Y352) is a nuclear serine/threonine kinase that modulates transcription factor activity and integrates stress and signaling pathways with gene expression regulation [43]. The clustering of predicted upstream kinases around S944 suggests that this site functions as a major phospho-regulatory hotspot positioned within or near a functional motif in the KH-domain architecture, potentially integrating MAPK, CDK, and stress-responsive signaling pathways to regulate HDLBP activity and cellular stress responses.

Protein interactors provide important insight into functional associations and regulatory mechanisms underlying HDLBP signaling. Analysis of binary interaction partners in this study revealed site-specific interaction networks associated with HDLBP phosphorylation at S31 and S944, supporting the involvement of these sites in distinct regulatory pathways. Several of these interactions have been previously reported, including the well-established HDLBP–BRAF interaction. In hepatocellular carcinoma, HDLBP was shown to physically interact with BRAF and inhibit its ubiquitination-mediated degradation, thereby promoting epithelial–mesenchymal transition (EMT) and metastasis [28].

HDLBP has been implicated in several key cellular functions, including RNA binding and translation of ER-targeted mRNAs [19], heterochromatin formation, chromosome segregation, nuclear transport [21,60], and stress–granule-related responses through interactions with TSC2 and other RNA-regulatory proteins [61]. The protein-level enrichment also revealed marked over-representation for chromatin remodeling, RNA metabolism, and nucleocytoplasmic transport, suggesting involvement in gene regulatory processes, nuclear organization, and cytoskeletal structure. Major proteins that showed enrichment in chromosome organization were WAPL, PDS5B, etc. WAPL is a regulator of cohesin release factor and PDS5B, a regulatory subunit of the cohesin complex involved in chromosome segregation and organization [62,63]. NUP133, NUP153, NUP214, and NUP35 are core components of the nuclear pore complex (NPC), which mediates nucleocytoplasmic transport of RNA and proteins. These nucleoporins contribute to nuclear pore assembly, mRNA export, protein import/export, and nuclear envelope organization [64,65]. The heterogeneous nuclear ribonucleoproteins (hnRNPs) including HNRNPA1, HNRNPA2B1, HNRNPM, and HNRNPH3 are key RNA-binding proteins involved in RNA metabolism, such as alternative splicing, mRNA stability, and translational regulation, while YTHDC1, RBM15, etc., further contribute this network to splicing and co-transcriptional RNA processing [66,67,68]. The site-specific-level functional mapping of the co-regulated proteins was found to participate in diverse cellular processes, including regulation of the cell cycle, apoptosis, inhibition of autophagy, transcriptional control, DNA repair mechanisms, modulation of signaling pathways, and reorganization of the cytoskeleton.

In addition to its well-characterized functions in RNA metabolism, HDLBP also plays significant roles in lipid metabolism and cholesterol transport. Consistent with these known functions, our analysis also revealed several co-regulated proteins involved in lipid homeostasis. The cellular cholesterol levels are mainly regulated by SCAP, SREBP, and INSIG. When cholesterol levels are high, these proteins form a complex that anchors to the endoplasmic reticulum (ER), which acts as a cholesterol sensor [69,70]. When cellular cholesterol levels are low, the SCAP–SREBP–INSIG complex dissociates and the SCAP–SREBP complex is then transported to the Golgi apparatus, where SREBP undergoes proteolytic cleavage by SCAP to become active. The activated SREBP then translocates to the nucleus, where it promotes the transcription of key genes involved in cholesterol and fatty acid biosynthesis, including HMGCS1, FASN, and SCD [71,72,73]. Although SREBP and INSIG were detected in the initial datasets, they did not meet the cutoff criteria. This may be because acetylation/deacetylation is the major post-translational modification involved in regulating SREBP, which could be the reason for the limited number of phosphorylation sites being detected [13,74]. In addition, the transcriptional co-activators of CREB responsive lipogenic genes, such as CREB1, CRTC2, CRTC3, and EP300, were found among the co-regulated proteins [75,76,77]. Furthermore, several proteins involved in lipid transport and intracellular trafficking were found to be co-regulated with HDLBP, including OSBPL8, OSBPL3, BLTP1, SEC14L1, and PNPLA6 [78,79,80,81]. Among these, BLTP1 is known to mediate non-vesicular lipid transport [82]. In addition, vesicular trafficking proteins such as SORT1, VPS35, SNX17, and PIKFYVE were also identified [83,84,85]. In short, our results are consistent with previous studies implicating the role of HDLBP in lipid metabolism, cholesterol biosynthesis, and intracellular trafficking.

Although HDLBP has been implicated in cancer, the functional role of its phosphorylation has not been explored. Consistent with this, analysis using the UALCAN platform revealed differential expression of HDLBP and its predominant phosphorylation sites across cancer types, implying a possible conserved role in tumor-related pathways. Specifically, HDLBPs were found to be differentially expressed in breast cancer, colon cancer, pancreatic carcinoma, glioblastoma, and HCC. Earlier studies reported that HDLBP expression can be downregulated or mutated in breast cancer, suggesting a tumor-suppressive role. Somatic mutations in HDLBP were identified in human breast tumors, implying loss-of-function effects that may disrupt its RNA-regulatory or lipid-transport roles [86]. In HCC, HDLBP is significantly upregulated and promotes tumor proliferation, metastasis, and sorafenib resistance by stabilizing RAF1 and maintaining MAPK signaling activity [27]. Although the molecular mechanisms remain incompletely defined, HDLBP likely functions as an oncogenic driver in cancers such as hepatocellular carcinoma and malignant mesothelioma, while in others, such as breast cancer, it may exert tumor-suppressive functions depending on the cellular and metabolic context [18].

Over the past two decades, phosphoproteomics has become a powerful tool for mapping signaling networks, generating large-scale datasets that reveal diverse phosphorylation patterns across biological and disease conditions [2]. Recent studies emphasize that understanding the functional roles of individual phosphorylation sites is critical for interpreting signaling regulation [87]. In this context, our in silico methodology offers a new framework for interpreting phosphorylation events and signaling patterns, advancing understanding of their biological relevance.

## 4. Materials and Methods

### 4.1. Assembly and Analysis of Global Phosphoproteomic Datasets with HDLBP Phosphosites

To identify high-confidence HDLBP phosphosites, a comprehensive PubMed search was performed using the keywords “phosphoproteomics” OR “phosphoproteome” and excluded the studies with plants or review articles. From the screened global phosphoproteomic datasets from human cells and tissues, we identified high confidence class-1 phosphosites with a localization probability ≥75% and an A-score ≥13. In phosphoproteomics, localization probability represents a statistical confidence score used to assign a phosphorylation event to a specific residue within a peptide when ambiguity exists in mass spectrometry data. This score estimates how confidently the phosphorylation can be localized to a particular serine, threonine, or tyrosine residue. Phosphosites with localization probability ≥75% are commonly designated as class-1 sites, indicating high-confidence site assignment and suitability for downstream biological interpretation. Similarly, the ambiguity score (A-score) provides an independent metric to quantify confidence in phosphorylation site localization. An A-score greater than 13 is likewise used to define class-1 phosphosites with high localization confidence. In this study, localization probability and A-score values were extracted from the supplementary data of published phosphoproteomics studies. Given the large scale of the compiled data (~3000 datasets), these widely accepted thresholds were applied uniformly across studies to retain only confidently localized phosphosites for subsequent analyses, thereby ensuring data reliability and comparability across datasets.

For subsequent analysis, the datasets were categorized based on the type of phosphosite enrichment methods (STY, ST, or Y). Additionally, the data sets were organized into quantitative differential profile datasets, which compared phosphosite abundances between experimental conditions and their respective controls, and qualitative profile datasets, where test conditions and controls are treated as independent phosphosite profiles.

The class-1 phosphosites of HDLBP are classified into upregulated and downregulated based on their fold change and *p*-value (<0.05). The phosphosites whose fold change value is ≥1.3 are selected as upregulated and ≤0.76 are selected as downregulated. Each protein was mapped into its corresponding gene symbol based on the HGNC (downloaded on 30 May 2023). Subsequently, the individual phosphosites in each of the datasets were mapped to their corresponding UniProt (13 April 2023) [88] accessions using our in-house mapping tool to ensure uniform mapping. The annotated biological and experimental conditions were tagged to each dataset in a standard format for efficient categorization [89]. We followed the methodologies followed in Pahal et al. for conducting the analysis [49].

### 4.2. Identification of Predominant Phosphosites of HDLBP

The phosphosites of HDLBP in the human cellular phosphoproteome profile datasets were assembled and analyzed. To identify the predominant phosphosite, the number of qualitative profile datasets where each phosphosite was detected and the number of quantitative profile datasets showing differential regulation was calculated and ranked. The predominant HDLBP phosphosites were selected primarily based on the differential frequency (how frequently they were observed) in quantitative datasets to investigate the CPPs. Phosphosites identified using specific phospho-antibodies or mutation-based approaches, but not frequently reported as class-1 sites in these datasets, are not accounted for further analysis.

### 4.3. Sequence Coverage and Peptide Map Analysis of HDLBP

To represent and visualize the peptide sequence, post-translational modifications (PTMs) on serine (S), threonine (T), and tyrosine (Y) residues, and the overall sequence coverage of the HDLBP protein, we analyzed the total sequence coverage and its class-1 phosphosites obtained from global human cellular phosphoproteomic profile data. This analysis focused on identifying the unique and frequently modified HDLBP peptide sequences, particularly those containing multiple PTMs across diverse biological contexts. The Sequence Coverage Visualizer (SCV), a protein structure prediction tool, was used to visualize sequence coverage and class-1 phosphosites [36].

### 4.4. Analysis of Phosphosites of Other Proteins That Are Co-Differentially Regulated with HDLBP Predominant Sites

To analyze the phosphosites of other proteins that are positively or negatively co-differentially regulated with the predominant phosphosites of HDLBP, the differentially regulated datasets from diverse experimental conditions, biological systems, and methodologies were categorized separately. Given the large number of datasets, reanalyzing the raw data was impractical. Instead, we classified datasets based on the regulation patterns of each predominant site upregulated (U) or downregulated (D) and examined the differential expression based on the previous methodologies established in our laboratory [49,90].

For each predominant site, CPPs were grouped based on their co-regulation patterns as UU (both upregulated), DD (both downregulated), UD (predominant site upregulated, CPPs downregulated), and DU (predominant site downregulated, CPPs upregulated). CPPs found consistently in UU and DD conditions were considered positively co-regulated (UUDD), while those in UD and DU categories were considered negatively co-regulated (UDDU).

To avoid any potential bias that could occur due to over-representation of biological data, we applied multiple stringent criteria for the selection of co-regulated proteins for further analysis. A one-sided Fisher’s exact test (FET) was employed across the datasets by constructing contingency tables to calculate the statistical significance between specific phosphosites and experimental conditions. The phosphosite pairs with an FET *p*-value < 0.05 that fall into the UUDD or UDDU categories of co-regulation with the predominant phosphosites were considered. For positively co-regulated CPPs, the ratio was calculated by the equation ∑(nUU + nDD)/∑(nUD + nDU), and the ratio for negative co-regulation was calculated using ∑(nUD + nDU)/∑(nUU + nDD). CPPs with a co-regulation ratio greater than 10% of the total frequency of the predominant HDLBP phosphosites were considered as high-confidence co-regulation. We applied additional filtering criteria, wherein we considered CPPs only if they appeared in at least three PubMed-indexed studies (PMID confidence ≥ 3) and were identified across three or more independent experimental conditions (experimental code confidence ≥ 3). CPPs that satisfied these filtering criteria were classified as high-confidence, or highly co-regulated proteins.

### 4.5. Co-Occurrence Analysis of HDLBP-Predominant Sites

A co-occurrence analysis was conducted to identify the mutual associations and co-regulation patterns of phosphosites within HDLBP, where we particularly identified the co-differential regulation patterns of phosphosite pairs within HDLBP. Each differential data set having multiple phosphosites of HDLBP detected in the same experimental condition was extracted. For each pair, we separately calculated the UU, UD, DD, and DU frequencies. The positive (n_UUDD) and negative (n_UDDU) co-regulation frequencies among the co-occurring phosphosites were calculated as stated above. This method allowed us to analyze potential interdependence between phosphorylation sites and to determine their functional proximity in co-regulation dynamics. A heat map to visualize co-occurrence of phosphosites was generated using the matplotlib and seaborn libraries in Python (Version 3.14) [91], where a positive or negative co-occurrence with a frequency above 3 is colored in violet or green gradients, respectively. The phosphosite that occurs more frequently (>3) was considered to be co-occurring or having similar functions.

### 4.6. Analysis of Potential Upstream Kinases and Interactors of HDLBP

The upstream kinases of HDLBP were identified by various bioinformatics-based prediction tools such as NetworKIN [92] and Automatic Kinase-specific Interactions Detection (AKID) (downloaded June 2023) [93] and based on the synthetic peptide screening for the assessment of the substrate specificity of the kinome, with a cutoff of the 90th percentile as reported in Johnson et al. (2023) [42]. Additionally, the co-regulated network of kinases and phosphatases was determined through HGNC [94] and co-phosphorylation-based kinase–substrate interaction prediction (CoPhosK) [95].

The interactors of HDLBP were extracted from different databases. The experimentally known protein–protein interactors were extracted from Human Protein Reference Database (HPRD) [96], Biomolecular Interaction Network Database (BIND) [97], Biological General Repository for Interaction Datasets (BioGRID) [98], and ConsensusPathDb release 35 [99], CORUM [100], and RegPhos 2.0 [100,101].

### 4.7. Pan-Cancer Analysis of HDLBP

Protein expression data for HDLBP were retrieved from the Clinical Proteomic Tumor Analysis Consortium (CPTAC) [102], which provides high-throughput, mass spectrometry-based proteomic profiles across multiple cancer types. To further assess HDLBP expression in a pan-cancer context, UALCAN, an interactive web resource integrating CPTAC and TCGA datasets was utilized. UALCAN enables comparative analysis of gene and protein expression, promoter methylation, and survival outcomes across diverse malignancies [103]. In this study, log2-normalized expression values were analyzed using UALCAN’s pan-cancer module to evaluate cancer-type–specific variations in HDLBP expression, with statistically significant differences identified using a *p*-value cutoff of 0.05.

### 4.8. Data Visualization Tools

The functional gene enrichment analysis of co-regulated proteins with HDLBP predominant phosphosites was performed using ShinyGO (v.0.85.) [104]. Cytoscape (3.10.3) [105], Path Visio 3 [105,106], Rawgraph (2.0), and BioRender (2023) were used for the visualization of the results and pathways. The UALCAN database was used to visualize the protein expression data obtained from the CPTAC database [103].

## 5. Conclusions

This study presents the first site-specific phosphoproteomic analysis of HDLBP, identifying S31 and S944 as predominant phosphorylation sites and revealing phosphorylation as a key regulatory layer in this multifunctional RNA-binding protein. Our analyses link HDLBP phosphorylation to RNA metabolism, chromatin organization, cytoskeletal regulation, and lipid homeostasis through co-ordinated kinase regulation and protein interaction networks. Differential regulation of HDLBP phosphorylation across multiple cancers further suggests context-dependent roles in tumor-associated signaling. Together, these findings provide a framework for future functional analyses and emphasize the need for experimental validation of HDLBP’s mechanistic roles.

### Limitations and Future Directions

Despite the robustness of our analyses, several limitations must be acknowledged. The study relies on computational analyses of existing phosphoproteome datasets; though comprehensive, these analyses still limit definitive inference. Thousands of phosphosites have been reported across the human proteome, yet most of them lack experimentally validated, site-specific functional annotations, and HDLBP is no exception. Generating such evidence through wet-laboratory experiments is technically demanding, expensive, and extremely time-consuming, especially when multiple phosphosites, kinases, and interacting partners need to be systematically tested. As a result, comprehensive phosphosite-level characterization is often impractical to pursue experimentally.

In this context, integrative computational approaches like ours play a crucial role, offering a scalable strategy to prioritize functionally relevant phosphosites, predict upstream kinases, and highlight biologically meaningful networks. Our findings necessitate experimental validation; however, they establish a robust and logical basis for forthcoming targeted studies focused on elucidating the functional implications of HDLBP-prioritized phosphorylations, especially at S31 and S944. Future works should therefore focus on targeted experimental strategies, including site-directed mutagenesis to generate phospho-deficient and phospho-mimetic variants; in vitro and cellular kinase assays to confirm upstream kinase-substrate relationships; kinase inhibition or activation studies to assess signaling dependency; co-immunoprecipitation and proximity-based interaction assays to validate phosphorylation-dependent protein interactions; and functional assays such as RNA-binding, localization, and splicing analyses to determine downstream regulatory effects. Collectively, such approaches would enable direct validation of the computational predictions and clarify the biological significance of HDLBP phosphorylation events.

## Figures and Tables

**Table 1:** Summary of the predominant phosphosite expression in different cancers.

Cancer Type	Phosphosites	*p*-Value
Breast cancer	S944	5.25 × 10^−5^
Colon cancer	S31	3.09 × 10^−2^
Colon cancer	S944	2.55 × 10^−19^
Pancreatic cancer	S944	5.30 × ^−5^
Glioblastoma	S944	1.49 × 10^−16^
Hepatocellular carcinoma	S31	3.31 × 10^−12^

## Data Availability

The original contributions presented in this study are included in the article and Supplementary Materials. Further inquiries can be directed to the corresponding author.

## Supplementary Materials

The following supporting information can be downloaded at: https://www.mdpi.com/article/10.3390/ijms27052147/s1.
